# Role reversal in a predator–prey interaction

**DOI:** 10.1098/rsos.140186

**Published:** 2014-10-01

**Authors:** Faustino Sánchez-Garduño, Pedro Miramontes, Tatiana T Marquez-Lago

**Affiliations:** 1 Facultad de Ciencias, Universidad Nacional Autónoma de México, México DF., 04510, Mexico; 2 Centro de Ciencias de la Complejidad, Universidad Nacional Autónoma de México, México DF., 04510, Mexico; 3 Integrative Systems Biology Unit, Okinawa Institute of Science and Technology, Onna-son, Okinawa 904-0412, Japan

**Keywords:** predator–prey, bistability, excitable media, reaction–diffusion

## Abstract

Predator–prey relationships are one of the most studied interactions in population ecology. However, little attention has been paid to the possibility of role exchange between species, despite firm field evidence of such phenomena in nature. In this paper, we build a mathematical model capable of reproducing the main phenomenological features of role reversal in a classical system and present results for both the temporal and spatio-temporal cases. We show that, depending on the choice of parameters, our role-reversal dynamical system exhibits excitable-like behaviour, generating waves of species' concentrations that propagate through space. Our findings fill a long-standing gap in modelling ecological interactions and can be applicable to better understanding ecological niche shifts and planning of sustainable ecosystems.

## 2. Introduction

Predator–prey interactions have been widely studied in the last hundred years, and their mathematical models are among the oldest established in ecology. One of the classic ecological models, the Lotka–Volterra model, was independently proposed by Alfred J. Lotka and Vito Volterra in the first quarter of the 1900s. Alfred J. Lotka developed preliminary models with a focus on demography, but later refined his model while turning his attention to trophic interactions by using a plant species and a herbivorous animal as an example. Vito Volterra, on the other hand, is said to have developed his version of the model by analysing fishery patterns in the Adriatic Sea. The wide origins and scales of the questions that led to this classic model show its inherent flexibility and adaptability, a probable reason why Lotka–Volterra models remain popular to this day.

Over the years, many modifications have been made to the original Lotka–Volterra model, to adapt to more realistic physical scenarios. Some examples are competition, mutualism [1] and generalization to several interacting species. However, so far, little has been done in generating models where the role of interacting species can change in time. Models of this sort are essential, as the ecological role of individual species may not be fixed, nor clear [2].

Role-exchanges, or role-reversals, are actually more common in nature than once believed. For instance, prey species may confront predators in a size-dominant manner [3–6], size-recessive manner [7], by changing population densities [8], or without specific trophic intentions [9]. Also, it is known that adult prey of some species attack vulnerable young predators, where prey recognize the species of predators they were exposed to during juvenile stages, and effectively attack their offspring [2,10].

So, the goal of our work was to fill this gap and propose a model that could account for role-reversal interactions. For that purpose, we focused on representing one of the classic references in the literature, the work by Barkai and McQuaid. However, it is important to note that the definition of interaction (trophic, sexual and hierarchical) can be easily modified once a model is constructed and, thus, our work is not solely limited to interactions of one type.

In 1988, Barkai & McQuaid [8] reported a novel observation in population ecology while studying benthic fauna in South African shores: a predator–prey role reversal between a decapod crustacean and a marine snail. Specifically, in Malgas Island, the rock lobster *Jasus lalandii* preys on a type of whelk, *Burnupena papyracea*. So, as could be easily expected, the population density of whelks soared upon lobsters' extinction in a nearby island (Marcus Island, just 4 km away from Malgas). However, in a series of very interesting controlled ecological experiments, Barkai and McQuaid reintroduced a number of *J. lalandii* in Marcus Island to investigate whether the equilibrium observed in the neighbouring Malgas Island could be restored. The results were simply astounding:

‘The result was immediate. The apparently healthy rock lobsters were quickly overwhelmed by large number of whelks. Several hundreds were observed being attacked immediately after release and a week later no live rock lobsters could be found at Marcus Island.’ [8, p. 63]

Surprisingly, and despite observations such as the report in [8], theoretical population biology has largely ignored the possibility of predators and preys switching their roles. Of importance, the paper of Barkai and McQuaid suggests the existence of a threshold control parameter responsible for switching the dynamics between (i) a classical predator–prey system with sustained or decaying oscillations, and (ii) a predator (the former prey) driving its present-day prey to local extinction.

In this paper, we build a model capable of reproducing this behaviour. It is worth noting that there are some papers in the literature describing ratio-dependent predation (e.g. [11,12]), but they are not related to the possibility of role-reversals. On the other hand, the likelihood of changing ecological roles as a result of density dependence has already been documented for the case of mutualism by Breton & Addicott [13] and, in 1998, Hernández and Barradas made an interesting effort to build a mathematical scheme capable of taking into account the possible switches among different possible ecological interactions [14]. So, to the best of our knowledge, this is the first theoretical study—supported by field evidence—specifically addressing predator–prey role-reversals.

## 3. Mathematical model

Predator–prey systems are generally modelled by adopting one of the many variations of the classical Lotka–Volterra model:

3.1 $$\begin{array}{rl} \dot{x} & =\alpha x-\beta xy\\ \text{and}\;\;\dot{y} & =-\gamma y+\delta xy, \end{array}\}$$

where *α* denotes the intrinsic preys' rate of growth, *β* corresponds to the rate of predation upon prey, *γ* stands for the predators' death rate in the absence of prey and *δ* represents the benefit of predators due to the encounters with prey. Our goal is to assess whether modelling the role-reversal behaviour observed by Barkai & McQuaid [8] is possible, when adopting appropriate parameters and assumptions.

For instance, if one considers quadratic density dependence in the prey as well as in the predators, non-constant rates of consumption of prey by the predators, and the profiting of predators by the existence of prey, then it is possible to suggest the following system:

3.2 $$\begin{array}{rl} \dot{x} & =Bx\left(\frac{A}{B}-x\right)-C(x)xy\\ \mathrm{and}\;\dot{y} & =-Dy-Ey^{2}+F(x)xy, \end{array}\}$$

where *B* represents the intrinsic growth rate of the prey in the absence of predators, *A*/*B* the carrying capacity of the prey's habitat, *C*(*x*) the rate of prey consumption by the population of predators, *D* the predators' decay rate in the absence of prey, *E* the intraspecific rate of competition among predators and, finally, *F*(*x*) the factor of predator's profiting from prey. The ratio *F*(*x*)/*C*(*x*) is then the fraction of prey biomass that is actually converted into predator biomass. The latter should remain constant, because the fraction of preys' biomass converted to predators' biomass is a physiological parameter, rather than a magnitude depending on demographical variables.

A particular case of system (2.2) in appropriate rescaled variables can be written as:

3.3 $$\begin{array}{rl} \dot{x} & =bx(1-x)-cx(k-x)y\equiv f(x,y)\\ \mathrm{and}\;\dot{y} & =-ey(1+y)+fx(k-x)y\equiv g(x,y), \end{array}\}$$

where all the parameters are positive and 0<*k*<1. In fact, all of the parameters have a relevant ecological interpretation: *b* is the normalized intrinsic growth rate of the species with density *x*, *c* is a measure of the damage intensity of the second species on the first one, *e* is the normalized rate of predators decay and *f* is the benefit (damage) the second population gets from the first one. Note the crucial role played by the interaction term *x*(*k*−*x*), where *k* stands for the first population threshold to switch from being prey to predator.

## 4. Phase portrait analysis

### 4.1 Nullclines and equilibria

After some basic algebra, it can be noted that the horizontal nullcline of the system of equations (2.3), that is [*b*(1−*x*)−*c*(*k*−*x*)*y*]*x*=0, has two branches: the vertical axis and the non-trivial branch

**Figure 1.**
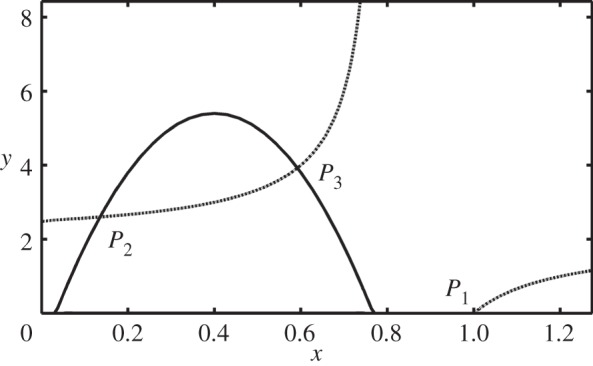
Nullclines of the system of equations (2.3). The continuous downward facing parabola and the horizontal axis are the vertical nullcline. All other lines, dotted and the vertical axis are the horizontal nullcline. *P*_1_, *P*_2_ and *P*_3_ are the non-trivial equilibria. The origin of coordinates is a trivial equilibrium.

4.1 $$y_{h}(x)=\frac{b(1-x)}{c(k-x)},$$

which is a symmetric hyperbola with asymptotes: *x*≡*k* and *y*=*b*/*c* (figure 1). The vertical nullcline, [−*e*(1+*y*)+*fx*(*k*−*x*)]*y*=0, also has two branches: the horizontal axis and the non-trivial branch

4.2 $$y_{v}(x)=\frac{fx(k-x)}{e}-1,$$

which is a parabola with *y*_*v*_(0)=*y*_*v*_(*k*)=−1, attaining its maximum at *x*=*k*/2, the value of which is

$$y_{v}\left(\frac{k}{2}\right)=\frac{fk^{2}}{4e}-1.$$

This term is positive if and only if *fk*^2^>4*e*. The zeros, *x*_1_ and *x*_2_, of equation (3.2) are given by

$$x_{1},x_{2}=\frac{f\pm \sqrt{f^{2}k^{2}-4fe}}{2f}.$$

The latter are real numbers if and only if *fk*^2^≥4*e*. The rate of change of *y*_*v*_ is then

$$y_{v}^{\prime }(x)=\frac{f}{e}(k-2x).$$

To analyse the system while keeping in mind the ecological interpretation of the variables and parameters, we will now consider the left branch of the horizontal nullcline (3.1), *fk*^2^>4*e* with 0<*k*<1 and the region of the phase plane of the system of equations (2.3) defined as

R={(x,y)∣0≤x≤1, 0≤y<+∞}.

The system of equations (2.3) has the equilibria: *P*_0_=(0,0), *P*_1_=(1,0) plus those states of the system stemming from the intersection of the nullclines *y*_*h*_ and *y*_*v*_ in the region R. Such equilibria are defined by the *x* in the interval (0,1) satisfying the identity

4.3 $$\frac{b(1-x)}{c(k-x)}=\frac{fx(k-x)}{e}-1,$$

or, equivalently, the *x* that are roots of the third-order polynomial

4.4 $$F(x)=Ax^{3}+Bx^{2}+Cx+D,$$

where *A*=*fc*, *B*=−2*fck*, *C*=*fck*^2^+*be*+*ec* and *D*=−*be*−*eck*.

The calculation of the non-trivial equilibria of equation (2.3) follows from the determination of the roots of equation (3.4). Consequently, owing to the qualitative behaviour of the functions *y*_*h*_ and *y*_*v*_ on R, we are faced with the following possibilities.

**Figure 2.**
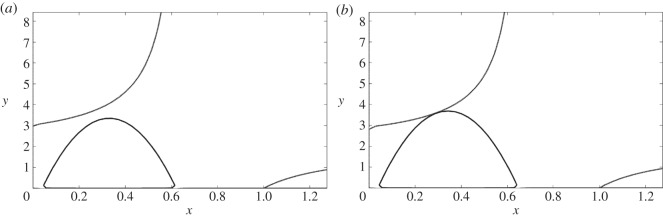
The relative position of the nullclines can be parametrically controlled. In (*a*), the downward facing parabola does not intersect the upper branch of the hyperbola. After a small change in the parameter *k*, both nullclines touch tangentially, as in (*b*). Further changes in the parameter lead to a saddle-node bifurcation and to the two transversal intersections depicted in figure 1.

1. The non-trivial branches of the nullclines do not intersect each other in the region of interest. In such a case, the system (2.3) has just two equilibria: *P*_0_ and *P*_1_ in R. Figure 2*a* shows the relative position of the nullclines in this case, and figure 3*a* the phase portrait of the system. For fixed positive values of *b*,*c*,*e* and *f*, and *k*∈(0,1) such that (*fk*^2^/4*e*)>1 one can see that both nullclines become closer with increasing values of *k*.
2. The nullclines *y*_*h*_ and *y*_*v*_ touch each other tangentially at the point P*=(x*,y*) in the region R. Again, figure 2*b* shows the relative position of the nullclines in this case, and figure 3*b* the phase portrait of the system. In such a case x*, in addition to satisfy equation (3.3), must also satisfy the condition *y*_*h*_′(*x*)=*y*_*v*_′(*x*), i.e.
  4.5 $$\frac{b}{c}\frac{\left(1-k\right)}{{\left(k-x\right)}^{2}}=\frac{f}{e}(k-2x).$$
  If one assumes the existence of x* satisfying equation (3.3), the required extra condition (3.5) imposes the restriction 0<x*<*k*/2 on x*, owing to the positiveness of its left-hand side. Moreover, from a geometrical interpretation of equation (3.5), it follows that, if:
  — 
    $$\frac{fk}{e}<\frac{b}{c}\frac{\left(1-k\right)}{k^{2}},$$
    there is not any *x*≥0 such that *y*′_*h*_(*x*)=*y*′_*v*_(*x*).
  — 
    $$\frac{fk}{e}=\frac{b}{c}\frac{\left(1-k\right)}{k^{2}},$$
    the condition (3.5) is satisfied just at *x*=0.
  — 
    $$\frac{fk}{e}>\frac{b}{c}\frac{\left(1-k\right)}{k^{2}},$$
    there exists exactly one value, x*∈(0,*k*/2), of *x*>0 such that the equality (3.5) holds.

**Figure 3.**
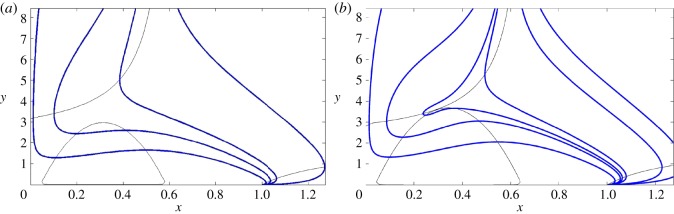
Phase portrait of the system. Blue/dark lines correspond to trajectories, whereas grey/light lines correspond to nullclines. When nullclines do not intersect, as seen in case (*a*), the origin is a saddle and there is only a non-trivial equilibrium on the *x*-axis, which is stable. All the initial conditions lead to lobsters extinction. However, when there is a tangential contact between the nullclines, as in case (*b*), there is a new non-hyperbolic equilibrium point, which is a saddle node.
  In any case, the point P* is a non-hyperbolic equilibrium of the system of equations (2.3). In fact, the proof that a tangential contact of the nullclines results in a point where the determinant of the Jacobian matrix of the system vanishes follows immediately, implying that at least one of its eigenvalues is zero.
3. The nullclines intersect each other transversally at two points, *P*_2_ and *P*_3_, belonging to the region R. For reference, please refer to figure 1. In this case, the system of equations (2.3) has two extra equilibria which arise from the bifurcation of P*. Here, if in addition to choosing the parameters *f*, *k* and *e* such that *fk*^2^/4*e*>1, we select the rest of them such that:
  — *y*_*h*_(*k*/2)>*y*_*v*_(*k*/2), i.e.
    $$\left[\frac{fk^{2}}{4e}-1\right]>\frac{b}{c}(2-k),$$
    guaranteeing the existence of the equilibria $P_{2}=({\tilde{x}}_{2},{\tilde{y}}_{2})$ and $P_{3}=({\tilde{x}}_{3},{\tilde{y}}_{3})$ mentioned above. Moreover, the coordinates of these points satisfy $0<{\tilde{x}}_{2}<k/2$, $k/2<{\tilde{x}}_{3}<k$, ${\tilde{y}}_{i}>0$ with ${\tilde{y}}_{2}<{\tilde{y}}_{3}$. Here, *i*=2,3.
  — *y*_*h*_(*k*/2)=*y*_*v*_(*k*/2), i.e.
    $$\left[\frac{fk^{2}}{4e}-1\right]=\frac{b}{c}(2-k).$$
    Here, we have $P_{2}=({\tilde{x}}_{2},{\tilde{y}}_{2})$ with $0<{\tilde{x}}_{2}<k/2$ and $0<{\tilde{y}}_{2}<(b/c)(2-k)$. Meanwhile, *P*_3_=(*k*/2,(*b*/*c*)(2−*k*)).

### 4.2 Local dynamics

Part of the local analysis of the system of equations (2.3) is based on the linear approximation around its equilibria. Thus, we calculate the Jacobian matrix of the system (2.3):

4.6 $$J{\left[f_{1},f_{2}\right]}_{\left(x,y\right)}=\left[\begin{array}{cc} b(1-2x)-cy(k-2x) & -cx(k-x)\\ fy(k-2x) & -e-2ey+fx(k-x) \end{array}\right].$$

By a straightforward calculation, we obtain the eigenvalues of the Jacobian matrix (3.6) at the point *P*_0_. These are: λ_1_=*b*>0 and λ_2_=−*e*<0. Hence, *P*_0_ is saddle point of the system (2.3), for all positive parameter values. By carrying out similar calculations, we obtain the corresponding eigenvalues of matrix (3.6) at *P*_1_, which are: λ_1_=−*b*<0 and λ_2_=*f*(*k*−1)−*e*. The restriction 0<*k*<1 on *k* implies that (*f*(*k*−1)−*e*)<0. Therefore, *P*_1_ is an asymptotically stable node for all the positive parameter values appearing in system (2.3).

Now we carry out the local analysis of (2.3). We note two cases, depending on the relative position of the nullclines.

*Case 1*. The main branches (3.1) and (3.2) of the nullclines do not intersect on R. Here, any trajectory of system (2.3) starting at the initial condition (*x*_0_,*y*_0_) with positive *x*_0_ and *y*_0_ tends to the equilibria *P*_1_ as time goes to infinity. Thus, the region $R^{+}$ is the basin of attraction of *P*_1_. Invariably, the species with density *y* vanishes, implying non-coexistence among the interacting species. Meanwhile, the other species approach the associated carrying capacity.

*Case 2*. The nullclines intersect each other at the points $P_{2}=({\tilde{x}}_{2},{\tilde{y}}_{2})$ and $P_{3}=({\tilde{x}}_{3},{\tilde{y}}_{3})$, where none is tangential. Here, ${\tilde{x}}_{2}$ and ${\tilde{x}}_{3}$ satisfy $0<{\tilde{x}}_{2}<k/2$ and $k/2<{\tilde{x}}_{3}<k$. In a neighbourhood of *P*_2_ and *P*_3_, the functions *f*_1_ and *f*_2_ satisfy the implicit function theorem. In particular, each one of the identities *f*_1_(*x*,*y*)=0 and *f*_2_(*x*,*y*)=0 define a function there. Actually, these are *y*_*h*_(*x*) and *y*_*v*_(*x*) given in equations (3.1) and (3.2), respectively. Their respective derivative at ${\tilde{x}}_{i}$ with *i*=2,3 is calculated as follows:

$$y_{h}^{\prime }({\tilde{x}}_{i})=-\frac{f_{1x}({\tilde{x}}_{i},{\tilde{y}}_{i})}{f_{1y}({\tilde{x}}_{i},{\tilde{y}}_{i})}\;\text{and}\;y_{v}^{\prime }({\tilde{x}}_{i})=-\frac{f_{2x}({\tilde{x}}_{i},{\tilde{y}}_{i})}{f_{2y}({\tilde{x}}_{i},{\tilde{y}}_{i})}.$$

By using these equalities, we can state the following proposition.

**Proposition 3.1**

*The equilibrium* *P*_2_ *is not a saddle point. Meanwhile, the equilibrium* *P*_3_ *is a saddle point for all the parameter values*.

A proof of this proposition and some remarks can be found in the electronic supplementary material, Appendix A.

### 4.3 Global analysis

**Figure 4.**
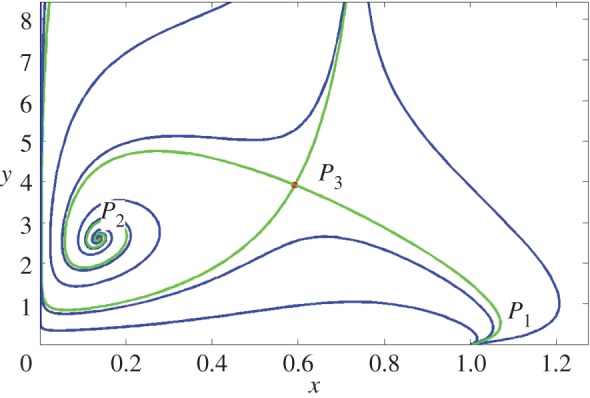
The equilibria of system (2.3). *P*_1_ and *P*_2_ are stable, whereas *P*_3_ is a saddle point. It is worth noting *P*_1_ is a node and *P*_2_ could be a node or a focus, depending on the parameter values. The heteroclinic trajectory joining the saddle point to the stable node is easily identified. The stable manifold is a separatrix between the basin of attraction of *P*_1_ and *P*_2_. The following parameters were used: *k*=0.8, *b*=0.6, *c*=0.3, *e*=0.05 and *f*=2.

As we have already shown, the system (2.3) could have up to four equilibrium points. These are illustrated in figure 4. The origin of coordinates is a saddle point with the horizontal and vertical axis as its unstable and stable manifolds. *P*_1_ and *P*_3_ are, respectively, a node and a saddle for parameter values after the bifurcation, and *P*_2_ could be a stable node. The stable manifold of the saddle point is a separatrix dividing the phase space in two disjoint regions: the set of initial conditions going to *P*_1_, and the complement with points going to *P*_2_. Moreover, our numerical solution shows the existence of a homoclinic trajectory starting and ending in the saddle point. Thus, we have a bistable system.

The bistability of system (2.3) has an interesting ecological interpretation: the coexistence of the interacting species occurs whenever the initial population densities (*x*_0_,*y*_0_) are located in the region above the saddle point unstable manifold. In this case, both populations evolve towards the attractor *P*_2_. On the other hand, if the initial population densities (*x*_0_,*y*_0_) are below the separatrix, the population densities (*x*(*t*),*y*(*t*)) evolve towards the equilibria *P*_1_, implying the non-coexistence of the species and, invariably, the species with population density *y* vanishes. The heteroclinic trajectory of system (2.3) connecting the saddle (*P*_3_) with the node (*P*_2_—or focus, depending on the set of parameters), in addition to the coexistence of the species, also tells us that this occurs by the transition from one equilibrium to another as time increases.

## 5. Spatial dynamics

To describe more accurately our role-reversal system, we extended our model of system (2.3) to incorporate the spatial variation of the population densities and confirm that our findings hold in spatial-explicit scenarios. Here, if we denote by *u*(***r***,*t*) and *v*(***r***,*t*), the population density of the whelks and lobsters at the point ***r*** at time *t*, the resulting model is

5.1 $$\begin{array}{rl} u_{t} & =D_{u}\nabla^{2}u+bu(1-u)-cu(k-u)v\\ \mathrm{and}\;v_{t} & =D_{v}\nabla^{2}v-ev(1+v)+fu(k-u)v, \end{array}\}$$

where the subscript in *u* and *v* denotes the partial derivative with respect to time, and ∇^2^ is the laplacian operator. Here, *D*_*u*_>0, *D*_*v*_>0 correspond to the diffusivity of the species with density *u* and *v*, i.e. that of whelks and lobsters, respectively. It is worth noting that the original variables have been rescaled, but still denote population densities.

We then proceeded to construct numerical solutions of the system (4.1) in three different domains: a circle with radius 2.2 length units (LU), an annulus defined by concentric circles of radii 2.2 and 1 LU, and a square with side length of 4.6 LU. All domains were constructed to depict similar distances between Malgas Island and Marcus Island (roughly 4 km). In the first one, the annular domain, we try to mimic the island habitat of whelks and lobsters as a concentric domain. The other two domains are used to confirm the pattern formation characteristic of excitable media and to reject any biases from the shape of the boundaries.

To obtain numerical solutions of all spatial cases, we used the finite-element method with adaptive time stepping and assumed zero-flux boundary conditions. Accordingly, we discretized all spatial domains by means of Delaunay triangulations, until a maximal side length of 0.17 LU was obtained. The latter defines the approximation error of the numerical scheme. We attempted to describe two entirely different situations by using a single set of kinetic parameters: that of Malgas Island, where both species coexist, and Marcus Island, where whelks soar and lobsters become extinct. The only difference between these two cases was the initial conditions used.

In terms of parameters, while there is no data specific to *J. lalandii* and *B. papyracea* in islands of the Saldanha Bay, data of similar species can be found in the literature. For instance, a related rock-lobster species, *Jasus Edwardii* has been found to move at a rate of 5–7 km d^−1^ [15]. By contrast, whelks within the superfamily *Buccinoidea* have been found to move towards food at rates between 50 and 220 m d^−1^ [16,17]. Importantly, predation by whelks remains seemingly unaffected by variations in water flow. In more detail, the statistical analysis in [18] showed no detectable differences between the total distances moved by whelks in reduced and enhanced flows. Moreover, whelk burrowing, prey searching and overall movement patterns were observed to be similar in both flow regimes. The likely reason for this is that whelks are slow moving, predatory gastropods that often forage with their bodies buried in the sediment and, among many key factors, they are less susceptible to flow-induced distortion of prey odour plumes. Of importance, the ranges of flow included in [18] were vast, matching conditions typical of benthic flows.

By putting these findings together, we argue a reasonable model need not incorporate influences from shallow water currents and would assume whelks to move towards ‘bait’ at a speed roughly one order of magnitude smaller than that of lobsters. Thus, we opted for a two-dimensional habitat, and one order of magnitude difference between the non-dimensional isotropic diffusion rates (*D*_*u*_=0.01 and *D*_*v*_=0.1). Aside, our choice of reaction parameters was: *b*=10, *c*=33.8, *e*=0.5, *f*=30 and *k*=0.9. Regarding initial conditions, we adopted the following scenarios, representing the different scenarios of weighted biomass.

1. Malgas Island: the initial density of whelks *u* at each element was drawn from a uniform distribution 0.1 * U(0.2,0.3), and that of lobsters *v* from U(0.2,0.3).
2. Marcus Island: the initial density of whelks *u* at each element was drawn from a uniform distribution U(0.2,0.3), and that of lobsters *v* from 0.1 * U(0.2,0.3).

**Figure 5.**
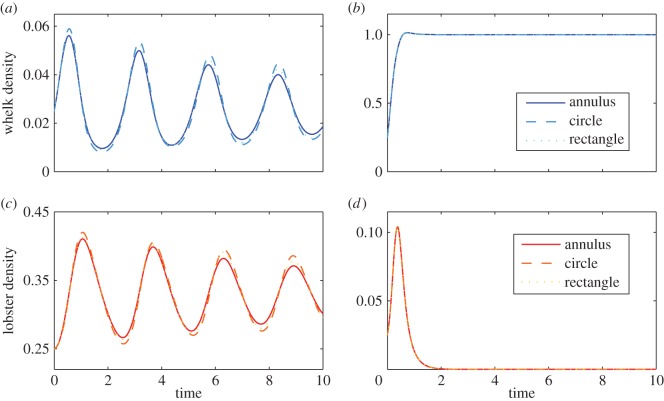
Time evolution of whelk and lobster densities, in different spatial domains. Cases (*a*,*c*) correspond to initial conditions representing Malgas Island, whereas (*b*,*d*) correspond to initial conditions representing Marcus Island.

The choice of reaction parameters was made upon a quick parameter sweep, leading to excitable-like behaviour. However, it should be noted many sets of parameters can be compatible with a given population dynamic. Regarding initial conditions, spatial simulations were performed with random initial conditions. In this way, it is possible to denote emergent patterns as robust with respect to variable initial conditions, and entirely independent of any pre-pattern. Numerical solutions of system (4.1) are shown in figure 5, corresponding to averaged densities of whelks and lobsters in the three different spatial domains, respectively. Movies of the corresponding simulations in an annular domain can also be found in the electronic supplementary material.

**Figure 6.**
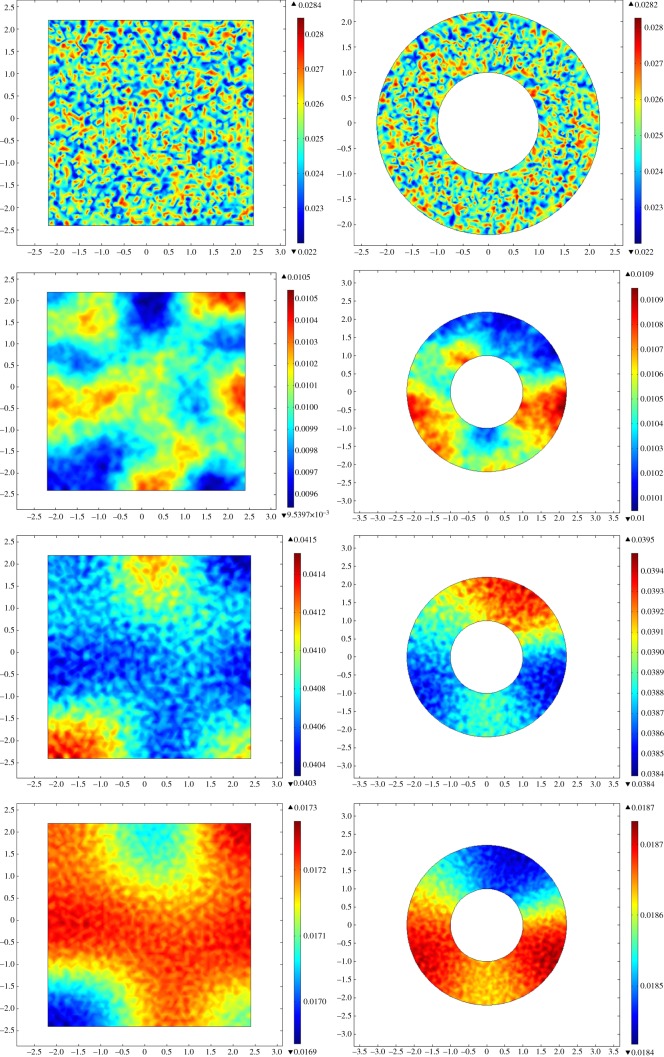
Time evolution of whelks' densities using initial conditions representing Malgas Island, in rectangular (left) and annular (right) domains. Cases correspond to *t*=0, 2, 6 and 10, from top to bottom. Whelk densities are colour coded, from high (red) to low (blue), within the density scales according to each time point.

**Figure 7.**
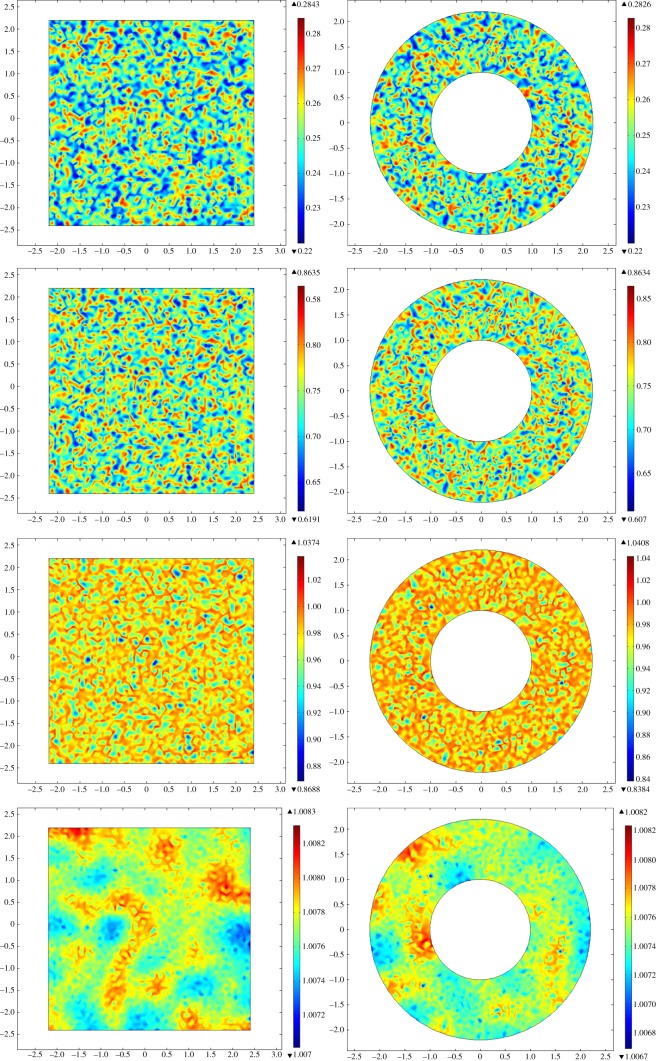
Time evolution of whelks' densities using initial conditions representing Marcus Island, in rectangular (left) and annular (right) domains. Cases correspond to *t*=0, 0.25, 0.5 and 1, from top to bottom. Whelk densities are colour coded, from high (red) to low (blue), within the density scales according to each time point.

Interestingly, changes in density are usually accompanied with wave-like spatial transitions in each species density. Examples of such spatial transient patterns can be found in figures 6 and 7, for annular and rectangular domains in Malgas Island and Marcus Island, respectively.

## 6. Discussion and final remarks

We have modelled a well-documented case of role-reversal in a predator–prey interaction, capturing the essential ecological factors within the study of Barkai & McQuaid [8], who did extraordinary fieldwork and meticulously reported this striking role-reversal phenomenon happening between whelks and lobsters in the Saldanha Bay.

The analysis of our model and corresponding numerical solutions clearly predict the coexistence of both populations and the switching of roles between the once denoted predators and prey. Here, the coexistence scenario corresponds to the case when lobsters predate upon whelks, and role-reversal corresponds to the case when whelks drive the population of lobsters to extinction, as observed by Barkai & McQuaid [8] in the field. However, it should be noted that in our model the switching of roles can occur for infinitely many combinations of the parameters. Specifically, the switching will occur when a state of the system crosses the separatrix, the unstable manifold of the saddle point in figure 4.

Moreover, by introducing spatial variables and letting both populations diffuse within a spatial domain, we obtain patterns that are characteristic of excitable media [19]. Of particular interest are the columns of figure 6, where self-sustained waves travel in both the rectangular and annular regions. The latter is not entirely surprising, as the ordinary differential equation model in which the spatial case was based shows bistability. Nevertheless, our findings are quite relevant in that, to the best of our knowledge, there are not many reports of ecological interactions behaving as excitable media so far. The only other work we are currently aware of is [20], where the authors model ocean plankton populations as excitable media.

Also, to the best of our knowledge, this is the first model proposed to both generate and represent role-reversals in ecological interactions, and may have wide applicability in explaining—and reverting—catastrophic shifts in ecosystems [21]. Importantly, role reversal was not introduced explicitly in our model and was only obtained as a consequence. In other words, our system was constructed using ecological first principles on the rate of change of the variables following field observations by Barkai and McQuaid, and role-reversal was only obtained as a result.

Lastly, it has been argued the creation of predictive models of role-reversal interactions will greatly alleviate efforts towards preventing ecological collapses or understanding alternative ecosystem states under changing conditions. The latter is owing to hypothesized path dependencies and ecosystem hysteresis [22]. Particularly, in marine ecosystems, internal feedback mechanisms have been proposed to be responsible for fundamental changes in ecosystem properties upon overfishing of predators [23]. Thus, a deeper, quantitative understanding of the roles played by species, and their possible exchanges, becomes essential. The latter will provide better foundation for selective harvesting, hunting and fishing strategies, leading to more sustainable and predictable ecosystems.

## Supplementary Material

Appendix A - Proof of Proposition

## Data accessibility

All additional data supporting this article, including mathematical proofs, have been uploaded as part of the supplementary material.
