# Supplementary material for: Role reversal in a predator–prey interaction
Source: R Soc Open Sci. 2014 Oct 1;1(2):140186. doi: 10.1098/rsos.140186 (PMC4448886; doi:10.1098/rsos.140186)
Supplement: Appendix A - Proof of Proposition [file rsos140186supp1.pdf]

Supplementary Material: Role reversal in a predator-prey interaction

August 19, 2014

1 Appendix A

Proof. First we prove the second part of our Proposition. At P_3 we have

$$y'_h(\tilde{x}_3) = -\frac{f_{1x}(\tilde{x}_3, \tilde{y}_3)}{f_{1y}(\tilde{x}_3, \tilde{y}_3)} > 0,$$

then the partial derivatives $f_{1x}(\tilde{x}_3, \tilde{y}_3)$ and $f_{1y}(\tilde{x}_3, \tilde{y}_3)$ have opposite signs at that point but $f_{1y} = -c\tilde{x}_3(k-\tilde{x}_3)$ with $k/2 < \tilde{x}_3 < k$ resulting in $f_{1y}(\tilde{x}_3, \tilde{y}_3) < 0$ hence $f_{1x}(\tilde{x}_3, \tilde{y}_3) > 0$. Similarly

$$y'_v(\tilde{x}_3) = -\frac{f_{2x}(\tilde{x}_3, \tilde{y}_3)}{f_{2y}(\tilde{x}_3, \tilde{y}_3)} < 0,$$

implying that f_{2x} and f_{2y} have the same sign at $(\tilde{x}_3, \tilde{y}_3)$ but $f_{2x}(\tilde{x}_3, \tilde{y}_3) = f(k-2\tilde{x}_3)\tilde{y}_3$ with $k/2 < \tilde{x}_3 < k$ and $\tilde{y}_3 > 0$, then $f_{2x}(\tilde{x}_3, \tilde{y}_3) < 0$ and $f_{2y}(\tilde{x}_3, \tilde{y}_3) < 0$. By using the above calculations we obtain $f_{1x}f_{2y} < 0$ and $f_{2x}f_{1y} > 0$ then, the determinant of the Jacobian matrix of the system (3) in the main text at P_3

$$detJ[f_1, f_2]_{(\tilde{x}_3, \tilde{y}_3)} = (f_{1x}f_{2y} - f_{2x}f_{1y}),$$

is negative. Therefore P_3 is a saddle point of (3) in the main text, for all the parameter values.

For the proof of the first part of the Proposition, we follow a similar sign analysis as we did previously, by considering that $0 < \tilde{x}_2 < k/2$, $\tilde{y}_2 > 0$ and that at $\tilde{x}_2$ the inequality $y'_h(\tilde{x}_2) < y'_v(\tilde{x}_2)$ holds where both derivatives are positive. Thus, given that $f_{1y}(\tilde{x}_2, \tilde{y}_2) = -c\tilde{x}_2(k - \tilde{x}_2)$ and $f_{2x}(\tilde{x}_2, \tilde{y}_2) = f(k - 2\tilde{x}_2)\tilde{y}_2$ the inequalities

$$f_{1y} < 0, \;\; f_{1x} > 0 \;\text{ and }\; f_{2x} > 0, \; f_{2y} < 0,$$

follow, from which we get $f_{1x}f_{2y} < 0$ and $f_{2x}f_{1y} < 0$. By using these inequalities and the condition $y'_h(\tilde{x}_2) < y'_v(\tilde{x}_2)$ one obtains

$$detJ[f_1, f_2]_{(\tilde{x}_2, \tilde{y}_2)} = (f_{1x}f_{2y} - f_{2x}f_{1y}) > 0,$$

with this we complete the proof. $\Diamond$

Remark 1. The trace of the Jacobian matrix (9) in the main text, at any point (x, y), is the quadratic

$$trJ[f_1, f_2]_{(x,y)} = -fx^2 + 2cxy + (fk - 2b)x - (ck + 2e)y + b - e. \qquad (1)$$

Given that its discriminant[1] $\Delta = AC - B^2 = -c^2 < 0$, (1) above is a quadratic equation of hyperbolic type. In order the see more details of such quadratic, we calculate its gradient. This is the zero vector at the point

$$(\hat{x}, \hat{y}) = \left(\frac{ck + 2e}{2c}, \frac{fe + bc}{c^2} \right) \in \mathcal{R}.$$

Given that the partial derivatives[2]

$$\frac{\partial^2 trJ}{\partial x^2}, \quad \frac{\partial^2 trJ}{\partial y \partial x} \quad \text{and} \quad \frac{\partial^2 trJ}{\partial y^2}$$

evaluated at any point (x, y) —in particular $(\hat{x}, \hat{y})$— are: $-2f < 0$, $2c > 0$ and 0 respectively, then we have

$$\left[\frac{\partial^2 trJ}{\partial x^2} \right] \left[\frac{\partial^2 trJ}{\partial y^2} \right] - \left\{ \frac{\partial^2 trJ}{\partial y \partial x} \right\}^2 = -4c^2 < 0.$$

Therefore $(\hat{x}, \hat{y})$ is a saddle point of the surface (1) above. The value of $trJ[f_1, f_2]$ at $(\hat{x}, \hat{y})$ is

If, for the parameter values we prove $trJ[f_1, f_2]_{(\tilde{x}_2, \tilde{y}_2)} < 0$ in addition to what we already have had proved, our conclusion follows.

[1]For the calculation of the discriminant we consider the general form of the quadratic

$$Ax^2 + 2Bxy + Cy^2 + Dx + Ey + F = 0,$$

hence $A = -f$, $B = c$ and $C = 0$.

[2]For notational convenience we simply write trJ instead of $trJ[f_1, f_2]_{(x,y)}$.
